# In vitro and in vivo assessment of the proresolutive and antiresorptive actions of resolvin D1: relevance to arthritis

**DOI:** 10.1186/s13075-019-1852-8

**Published:** 2019-03-12

**Authors:** Houda Abir Benabdoun, Merve Kulbay, Elsa-Patricia Rondon, Francis Vallières, Qin Shi, Julio Fernandes, Hassan Fahmi, Mohamed Benderdour

**Affiliations:** 1 Department of Pharmacology, Université de Montréal, Montreal,, QC Canada; 2 Orthopedic Research Laboratory, Hôpital du Sacré-Cœur de Montréal, Room K-3045, 5400 Gouin Blvd. West, Montreal, QC H4J 1C5 Canada; 3 Department of Surgery, Université de Montréal, Montreal, QC Canada; 4 Osteoarthritis Research Unit, Centre Hospitalier de l’Université de Montréal, Montreal, QC Canada; 5 Department of Medicine, University of Montreal, Montreal, QC Canada

**Keywords:** Arthritis, Inflammation, Resolvin D1, Bone resorption, Mice

## Abstract

**Background:**

Resolvin D1 (RvD1), an important member of resolvins, exerts a wide spectrum of biological effects*,* including resolution of inflammation, tissue repair, and preservation of cell viability. The aim of the present study is to investigate the anti-arthritic potential and clarify the bone protective actions of RvD1 in vitro and in vivo.

**Methods:**

RAW264.7 cells were treated with 50 ng/ml LPS for 72 h in the presence or absence of RvD1 (0–500 nM). Primary human monocytes were treated with M-CSF + RANKL for 14 days ± RvD1 (0–500 nM) with or without siRNA against RvD1 receptor FPR2. Expressions of inflammatory mediators, degrading enzymes, osteoclasts (OC) formation, and bone resorption were analyzed. The therapeutic effect of RvD1 (0–1000 ng) was carried out in murine collagen antibody-induced arthritis. Arthritis scoring, joint histology, and inflammatory and bone turnover markers were measured.

**Results:**

RvD1 is not toxic and inhibits OC differentiation and activation. It decreases bone resorption, as assessed by the inhibition of TRAP and cathepsin K expression, hydroxyapatite matrix resorption, and bone loss. In addition, RvD1 reduces TNF-α, IL-1β, IFN-γ, PGE_2_, and RANK and concurrently enhances IL-10 in OC. Moreover, in arthritic mice, RvD1 alleviates clinical score, paw inflammation, and bone and joint destructions. Besides, RvD1 reduces inflammatory mediators and markedly decreases serum markers of bone and cartilage turnover.

**Conclusion:**

Our results provide additional evidence that RvD1 plays a key role in preventing bone resorption and other pathophysiological changes associated with arthritis. The study highlights the clinical relevance of RvD1 as a potential compound for the treatment of inflammatory arthritis and related bone disorders.

## Introduction

Rheumatoid arthritis (RA) is a chronic immune-mediated inflammatory disease that features persistent inflammation leading to joint tissue destruction [1]. Afflicting up to 1% of the general population worldwide, women are three to five times more likely to develop RA than men [2, 3]. Highly debilitating, RA leads to impair joint function, in addition to severe pulmonary, renal, and cardiovascular dysfunctions, thereby affecting patients’ quality of life and significantly decreasing their life expectancy [3, 4]. Joint injuries are associated with excessive synovial inflammation and hyperplasia which promotes recruitment of inflammatory cells [5]. These activated cells will further induce excessive release of pro-inflammatory and catabolic mediators within the joint, thereby causing cartilage and bone breakdown [5–7].

Due to the central role of the immune system and inflammation in the pathogenesis of RA, current therapies mainly target inflammatory mediators such as tumor necrosis factor-alpha (TNF-α) and interleukin-6 (IL6) [8]. However, even if the currently available therapies such as disease-modifying anti-rheumatic drugs improve RA symptoms, they are effective in only half of the treated patients [9, 10]. Moreover, the severe side effects attributed to the long-term and high dosage usage limit their use [8]. Most importantly, none of these agents repair or even control joint damage [10, 11]. This underlines the importance of finding alternative pathophysiological pathways that could identify a new target leading, in terms, to a more adequate management of RA.

Hence, 2015 international recommendations for RA treatment set a dozen goals, the main one being to improve patients’ long-term quality of life, by reducing symptoms and preventing irreversible joint damage. They have pointed out not only controlling, but eliminating inflammation as the most effective way to achieve it [12]. Promoting the resolution of inflammation could be one effective way to reach this goal. Indeed, the identification of the resolution phase of inflammation as an active process, once believed to be passive, has highlighted the fact that there is a fine line between sufficiently strong inflammation and uncontrolled chronic inflammatory responses seen in inflammatory diseases such as RA. Failure in the resolution responses may therefore be responsible for that line being overpassed [13, 14].

The resolution process is orchestrated by a number of mediators such as resolvins [15]. They are derived from omega-3 polyunsaturated fatty acids, with well-described anti-inflammatory and proresolutive activities [16, 17]. By counter-regulating pro-inflammatory mediators and decreasing neutrophil recruitment in inflammatory sites, these mediators actively trigger the resolution of inflammation and promote the return to homeostasis [18, 19]. Resolvin D1 (RvD1), an important member of resolvins, is biosynthesized from the omega-3 docosahexaenoic acid (DHA; C22:6) via 15-lipoxygenase (15-LOX) and 5-LOX interactions in humans [20, 21]. RvD1 properties encompass a wide spectrum, ranging from potent anti-inflammatory and proresolutive actions to analgesic properties in inflammatory pain [20–23]. Moreover, we previously reported that RvD1 level is higher in knee synovial fluid from patients with osteoarthritis [24]. We, furthermore, demonstrated the potency of RvD1 in controlling inflammatory and catabolic responses, in human osteoarthritic chondrocytes.

Aiming to extend our research on the potential of RvD1 effects in inflammatory osteoarticular conditions, the purpose of this study is to determine the effects of RvD1 on RA onset and progression in vivo using a mouse model that shares many features with human RA as well as the molecular mechanism involved, using an in vitro model.

## Materials and methods

### Materials

RvD1, LDH ELISA kit, and PGE_2_ EIA kit were obtained from Cayman Chemical (Ann Arbor, Ml, USA). AMEM, RPMI 1640 1X medium, FBS, and antibiotics were purchased from Wisent Bio Products (Montreal, QC, Canada). LPS (*Escherichia coli* 0111:84), RANKL, M-CSF, TRAP staining kit, and mouse anti-β-actin antibody were obtained from Sigma-Aldrich (Oakville, ON, Canada). MTS assay kit was purchased from Promega Corporation (Madison, WI, USA). Primary antibodies against mouse TRAP and cathepsin K, von Kossa (calcium stain) kit, and rabbit polyclonal anti-Beclin-1 were obtained from Abcam Inc. (Toronto, ON, Canada). Peroxidase IgG secondary antibody was purchased from Jackson ImmunoResearch Laboratories (West Grove, PA, USA). TNF-α and IL-10 ELISA kits were purchased from R&D systems (Minneapolis, MN, USA). Th17-6 plex cytokine assay kit was purchased from Bio-Rad (Mississauga, ON, Canada). CTX-II ELISA kit and anti-mouse FPR2 antibody were purchased from MyBiosource (San Diego, CA, USA). CTX-I EIA kit was purchased from Immunodiagnostic Systems Limited (Boldon, UK). Ficoll-Paque PLUS was obtained from GE Healthcare (Mississauga, ON, CA). Osteo Assay Stripwell plates were purchased from Corning Inc. (New York, NY, USA). Arthrogen-CIA Arthrogenic Monoclonal Antibody was purchased from Chondrex (Redmond, WA, USA). FPR2 siRNA and scramble siRNA were purchased from Santa-Cruz Biotechnology (Santa-Cruz, CA, USA).

### Cell culture

Murine macrophage RAW 264.7 (ATCC, Manassas, VA, USA) were cultured with αMEM/10% FBS and antibiotics at 37 °C in a humidified atmosphere with 5% CO_2_. Primary human monocytes were isolated from whole blood obtained from healthy volunteers. Briefly, blood was centrifuged on a Ficoll-Paque density gradient, as described previously [25]. Isolated monocytes were then cultured in RPMI 1640 medium supplemented with 10% FBS, and antibiotics. All donors provided written, informed consent for the use of their blood for research purposes. Experimental protocols were approved by the Research Ethics Board of the “Hôpital du Sacré-Coeur de Montréal.”

### Animals

Thirty 8-week-old female DBA/1J mice, weighing approximately 18–20 g, were purchased from Jackson Laboratories (Bar Harbor, ME, USA). Animal handling and experimental procedures were conducted in compliance with the Canadian Council on Animal Care guidelines. The experimental protocol was adapted from previously reported methods [26] and approved by the Animal Research Ethics Committee of Hôpital du Sacré-Coeur de Montréal.

### Viability assay and LDH release

RAW 264.7 cells were cultured as described above then seeded in a 96-well plate at 4 × 10^4^ cells/well then treated with RvD1 (0–500 nM) with or without LPS (50 ng/ml) for 48 h. Cell viability and LDL release were assessed with commercial kits under the manufacturer’s instructions. The absorbance was measured at 590 nm with EL800 universal micro-plate readers (Bio-Tek Instruments, Winooski, VT, USA).

### TRAP staining

RAW 264.7 cells were cultured as described previously, seeded in chambered cell culture slides at 8 × 10^4^ cells/well, and transfected or not with 100 nM FPR2 siRNA or scramble siRNA. Osteoclast formation was induced by treatment of cells with LPS (50 ng/ml) ± RvD1 (0–500 nM) for 72 h. TRAP staining was performed as recommended by the manufacturer. Nuclei were counter stained with Gill’s hematoxylin and TRAP-positive multinucleated osteoclast staining (≥ 3 nuclei) was counted in 10 randomly selected high-power fields using digital EVOS light microscopy (Electron Microscopy Sciences, Hatfield, PA, USA) at × 20 magnification.

### Western blot

RAW 264.7 cells were seeded in a 24-well plate at 2 × 10^5^ cells/well then treated with RvD1 (0–500 nM) with or without LPS (50 ng/ml) for 72 h. Approximately 20 μg total proteins was loaded onto a 4–12% gradient SDS-PAGE and transferred to a nitrocellulose membrane (Bio-Rad Laboratories, Mississauga, ON, Canada). The primary antibodies were anti-mouse TRAP, anti-mouse cathepsin K, anti-mouse beclin-1, anti-FPR2, and anti-mouse β-actin primary antibodies. Revelation of immunoreactive bands and semi-quantitative analysis were performed as described in our previous report [24].

### TNF-α, IL-10, PGE_2_, and RANK quantification in cell culture supernatant

TNF-α, IL-10, and RANK levels were assessed in cell culture supernatants by ELISA, and PGE_2_ level was determined by EIA, according to the manufacturer’s instructions. All assays were performed in duplicate. The absorbance was quantified with the micro-ELISA Vmax photometer at 405 nm (Bio-Tek Instruments, Winooski, VT, USA).

### Hydroxyapatite resorption assay pit formation assay

Mononuclear cells were seeded in an Osteo Assay Stripwell plate at 5 × 10^5^ cells/well and allowed to adhere (2 h at 37 °C) in RPMI/10% FBS in a humidified atmosphere and 5% CO_2_. The nonadherent cells were removed by vigorous washes with PBS, and adherent cells (mainly monocytes/macrophages) were cultured in RPMI/10% FBS containing 10 ng/ml M-CSF and 50 ng/ml sRANKL. They were concurrently treated with increasing concentrations of RvD1 (0–500 nM) over 2 weeks. The culture medium was renewed every 3 days.

The resorption area in each plate was analyzed by von Kossa staining following the manufacturer’s instruction, then visualized and measured in 10 randomly selected high-power fields using digital EVOS light microscopy at × 20 magnification.

### Bone resorption in an ex vivo model

Bone explants from normal mice knee joints were rinsed in PBS containing antibiotics and cultured in BGJb medium supplemented with 20% FBS and antibiotics. Samples were treated or not with 10 ng/ml of M-CSF and 50 ng/ml of RANKL for 28 days in the presence or absence of 500 ng/ml RvD1. Culture medium were renewed two times per week. Bone histomorphometry was performed as described in our previous report [27] by measuring bone surface (μm^2^) and marrow cavity area (μm^2^).

### Collagen antibody-induced arthritis (CAIA)

Arthritis was induced using an arthritogenic cocktail of five monoclonal antibodies anti-type II collagen (mAb) combined to LPS as previously describe [26]. Briefly, on day 1, mice were immunized by an intra-peritoneal (i.p.) injection of 1.5 mg of mAb, followed on day 4 by an i.p. injection of 50 μg of LPS (*E. coli* 0111:84). Mice were monitored daily for body weight and disease scoring and then sacrificed on day 10. Animals were randomly separated into six groups: group 1, non-immunized mice; group 2, CAIA mice; groups 3–5, CAIA mice received a daily i.p. injection of 100, 500, and 1000 ng RvD1 since day 1, respectively; and group 6, CAIA mice received a daily i.p. injection of 1000 ng RvD1 at day 4. Groups 1 and 2 were given a saline solution with 0.1% EtOH instead. On day 10, mice were anesthetized by isoflurane inhalation, and blood was collected by cardiac puncture and then euthanized. Serum samples were stored at − 80 °C, and knees and ankles were conserved in formalin until analyzed.

### Clinical evaluation of induced arthritis

Arthritis onset and progression were monitored daily by recording hind paw thickness using a caliper, arthritic score, and body weight, by two independent observers blinded to treatment groups. The arthritic score was graded using a 4-point clinical index for each paw, based on macroscopic signs (0 = no evidence of inflammation, 1 = inflammation (swelling and/or redness) in one joint, 2 = inflammation in two joints, 3 = inflammation in more than two joints, and 4 = severe arthritis of the entire paw and joints) as described by Lee et al. [28], for a total score of 0–16 for all four paws.

### Serum inflammatory mediator’s determination

TNF-α, IL-17, IL-1β, IL-6, and IFN-γ were quantified in serum using a convenient bioplex kit assay, according to the manufacturer’s instructions. The concentrations of cytokines were measured using Bio-Plex™ 200 System instrument (Bio-Rad Laboratories, Hercules, CA, USA) and Bio-Plex manager 4.1 Software.

### Bone and cartilage remodeling assessment

CTX-I and CTX-II were quantified in mice serum by an EIA and ELISA kit, respectively, under the manufacturer’s instructions. The sensitivity of each kit is 4.5 and 10 pg/ml, respectively, and the absorbance was measured at 450 nm with EL800 universal micro-plate readers (Bio-Tek Instruments, Winooski, VT, USA).

### Histopathology study

Histopathologic sections of the knees were prepared following a conventional histopathologic processing of fixation, decalcification, and paraffin embedding, as we previously reported [29]. The joint sections were stained with safranin-O to assess cartilage degradation. Cartilage destruction was evaluated by two blinded observers, as described in detail in [29] using a previously described scoring system [30]. Osteoclast recruitment and activation were revealed by TRAP staining, as described in “TRAP staining” section. Cell proliferation in synovial membrane was performed by hematoxylin and eosin staining.

### Micro-CT (μ-CT) analysis

After the sacrifice, the ankles from each mice group were fixed with 4% paraformaldehyde for 24 h and placed in PBS for three-dimensional (3D) μ-CT analysis. Each sample was scanned using Skyscan 1176-High Resolution in vivo X-ray Microtomography (Bruker-MicroCT, Kartuizer Weg 3B, Kontich 2550, Belgium) at 50 kVp and 500 uA [26]. The 3D reconstruction was carried out using NRecon software, and the images were then after processed with μ-CT analyzer software.

### Statistics

All experiments were at least triplicated. Mean comparisons were performed by the analysis of variance (ANOVA) with appropriate post hoc analysis or two-way ANOVA with repeated measures where appropriate. Data are expressed as mean ± S.E.M. *p* values < 0.05 were regarded as significant.

## Results

### RvD1 does not affect murine RAW 264.7 macrophage viability

**Fig. 1:**
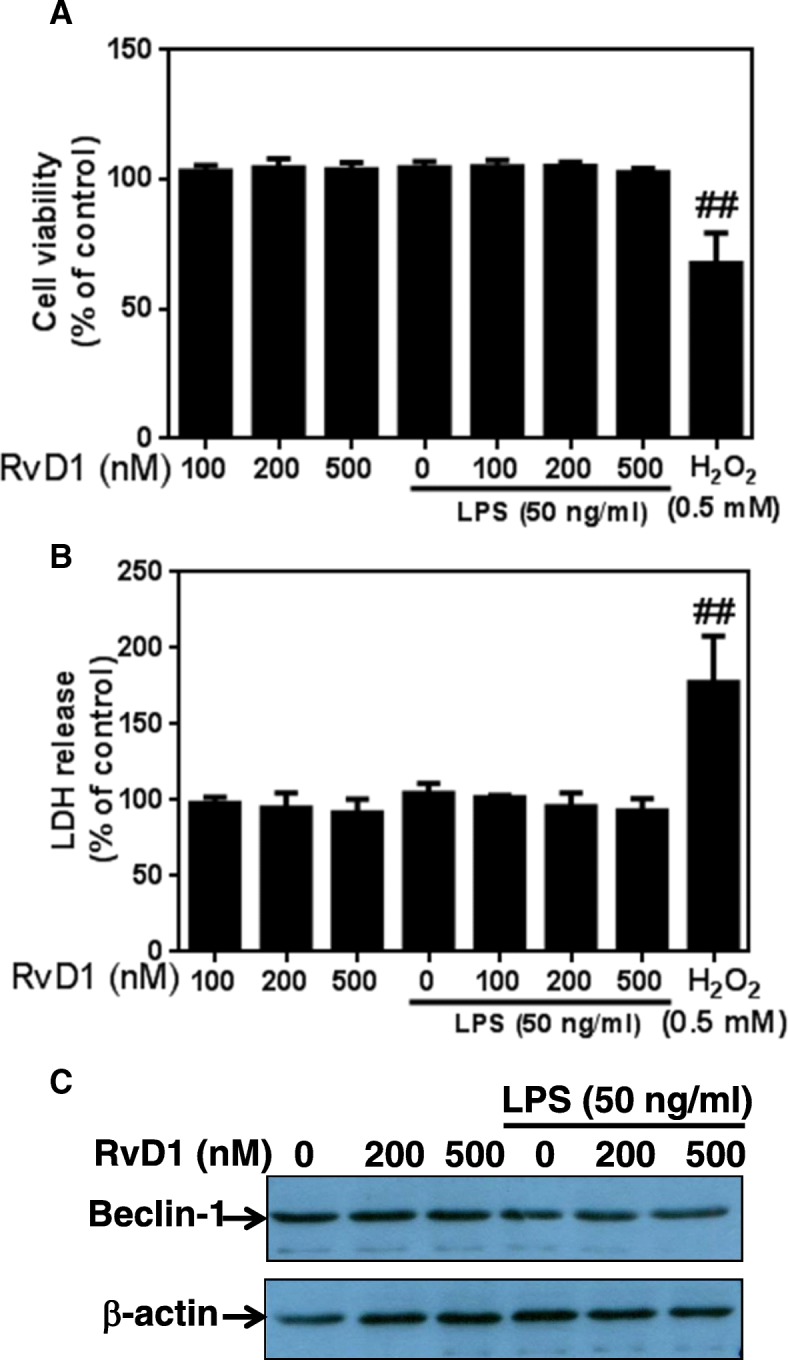
RvD1 does not impair RAW264.7 cell viability. RAW264.7 cells were incubated with different concentrations of RvD1 (0–500 nM) without or with 50 ng/ml LPS for 72 h. Cell viability percentages were obtained by 3-(4,5-dimethyl-thiazoyl)-2,5-diphenyl-SH-tetrazolium bromide assay (**a**), and LDH release was measured in cell supernatants by a commercial kit (**b**). Cell death and LDH release were induced by the addition of 0.5 mM H_2_O_2_. Beclin-1 protein expression was assessed in cell extracts by western blot (**c**). ANOVA test was performed to compare each condition. Results are expressed as mean ± SEM for *n* = 4. Data are means ± SEM, and one-way ANOVA was performed to compare the results. ^##^*p* < 0.01 compared to untreated cells

We first evaluated the potential toxicity of RvD1 in murine RAW 264.7 macrophages by performing an MTS assay. In contrast to H_2_O_2_, RvD1 did not alter the cell viability and LDH release after 48 h of incubation at tested doses ranging from 0 to 500 nM (Fig. 1a, b). Finally, the expression of Beclin-1, which is regulated in autophagy program cell death [31], was also unaffected by RvD1, in the presence or absence of LPS (Fig. 1c). Together, these data suggest that RvD1 does not affect cell viability.

### RvD1 attenuates osteoclast differentiation and inflammatory mediator expression in RAW264.7 macrophages

**Fig. 2:**
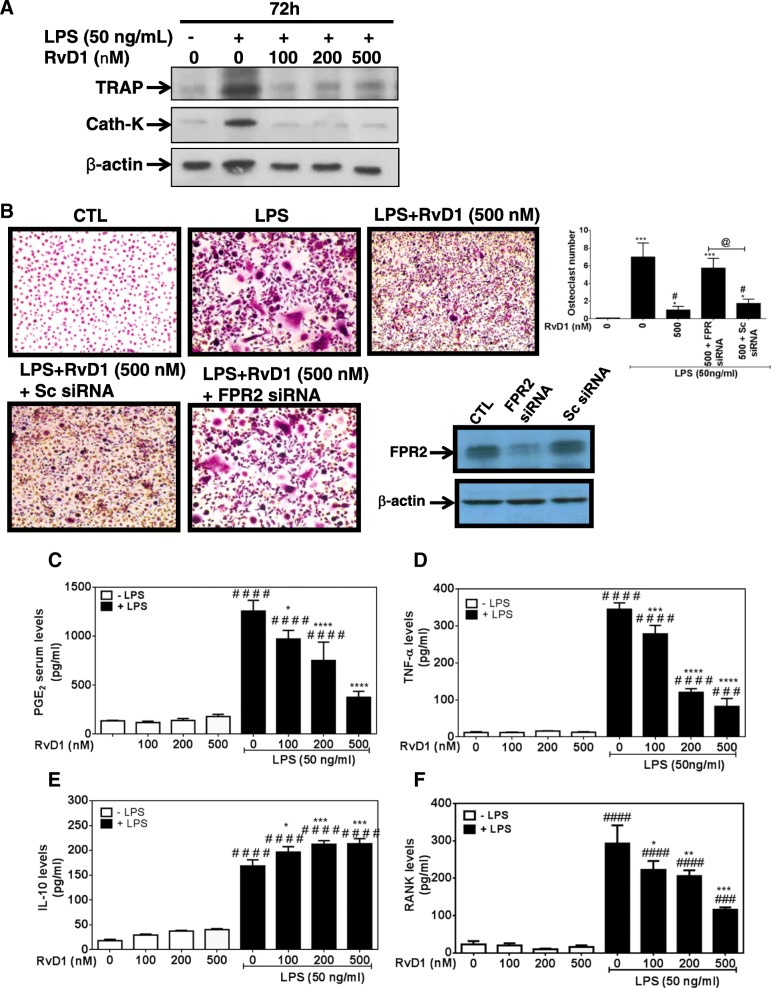
RvD1 inhibits osteoclast activation and recruitment as well as LPS-induced TRAP, cathepsin k, PGE_2_, TNF-α, and RANK expression and concurrently enhances IL-10 release in LPS-activated RAW 264.7 macrophages. Macrophages were stimulated with LPS (50 ng/ml) with or without RvD1 treatment (0–500 nM) for 72 h. Cell extracts were collected, and then TRAP and cathepsin k protein expression was assessed by western blot (**a**). The same treatment was conducted after incubation or not of cells with sc-siRNA or siRNA against FPR2 (50 nM) for 24 h. TRAP enzymatic staining was performed, and TRAP-positive cells were observed with an inverted microscope (× 200). Arrows show osteoclasts with three nuclei or more (**b**). Western blot was employed to confirm FPR2 silencing by siRNA (**b**). In parallel, cell media was collected and then PGE_2_ (**c**), TNF-α (**d**), IL-10 (**e**), and RANK (**f**) expression was assessed by commercial kits. Data are means ± SEM for *n* = 3, and one-way ANOVA was performed to compare the results. ^#^*p* < 0.05, ^##^*p* < 0.01, ^###^*p* < 0.001, and ^####^*p* < 0.0001 compared to non-stimulated cells; *p < 0.05, **p < 0.01, ***p < 0.001, and ****p < 0.0001 compared to LPS-activated cells

Here, we evaluated the impact of RvD1 treatment on RAW 264.7 macrophage-LPS-derived osteoclasts, by measuring their phenotypic markers, namely TRAP and cathepsin K. Western blot data show a strong inhibition of LPS-induced TRAP and cathepsin K expression by RvD1 at different concentrations (Fig. 2a) as well as TRAP activity and osteoclast differentiation (three to fourfold of inhibition, *p* < 0.05, Fig. 2b). Interestingly, downregulation of FPR2 expression by a specific siRNA abrogated the inhibitory effect of RvD1 as compared to LPS and LPS + scrambled (Sc) control siRNA (Fig. 2b). Of note, cell viability of transfected RAW264.7 with FPR2 siRNA or Sc siRNA was not affected and reached ~ 90–95% in comparison to untransfected cells.

We next investigated the anti-inflammatory of RvD1 in RAW 264.7 macrophage-LPS-derived osteoclasts. When used alone, RvD1 does not affect PGE_2_, TNF-α, and IL-10 releases neither RANK expression (Fig. 2c–f). In contrast, RvD1 significantly reduces LPS-induced PGE_2_, TNF-α, and RANK expression in a dose-dependent manner. At 500 nM, RvD1 significantly decreased LPS-induced PGE_2_, TNF-α, and RANK by three to fourfold (Fig. 2c, d, f; *p* < 0.001). In contrast, IL-10 level is significantly enhanced in RvD1-treated cells during LPS-induced osteoclast differentiation (Fig. 2e, *p* < 0.0001). Together, these results suggest that RvD1 inhibits osteoclast differentiation and activation, therefore reducing their pro-inflammatory mediator releases.

### RvD1 prevents bone erosion using primary human monocyte-derived osteoclasts

**Fig. 3:**
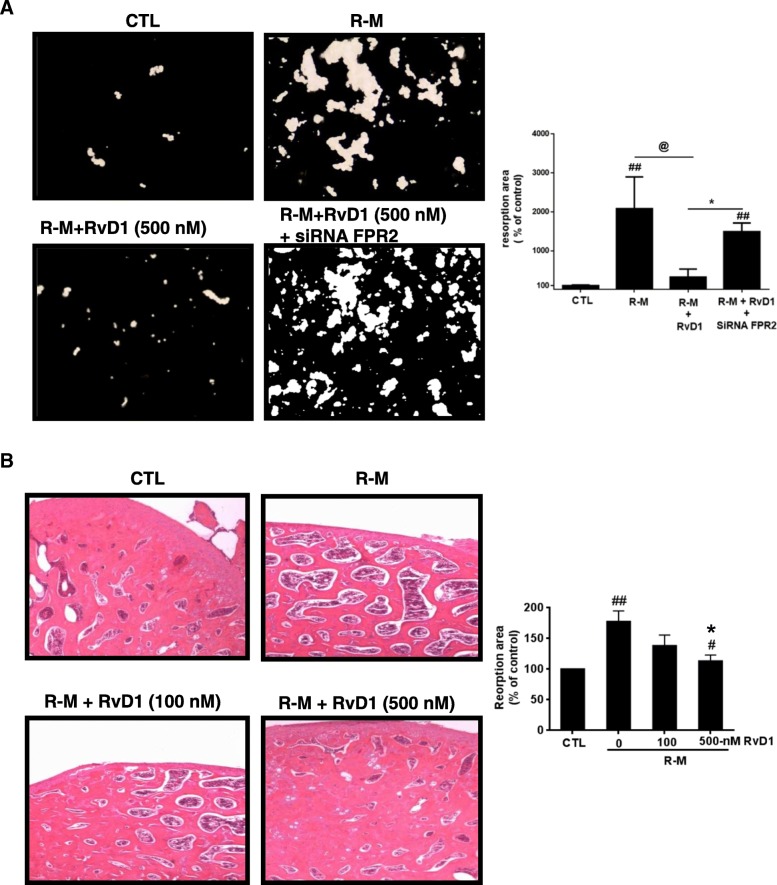
RvD1 inhibits hydroxyapatite matrix degradation as well as bone resorption induced by primary human monocyte-derived osteoclasts. **a** Human monocytes were incubated on a hydroxyapatite matrix. They were first treated without or with sc-siRNA or siRNA FPR2 (50 nM) for 24 h and then stimulated over 2 weeks with RANKL (50 ng/ml) and M-CSF (10 ng/ml) with or without RvD1 treatment (500 nM). Culture medium was changed every 3 days. Von Kossa staining was performed, then pit areas were observed under an inverted microscope (× 200) and measured using ImageJ software. **b** Normal mouse femoral bone explants were incubated with RANKL (50 ng/ml) and M-CSF (10 ng/ml) with or without RvD1 treatment (100 or 500 nM) over 28 days. Culture medium was changed every 3 days. Hematoxylin-eosin staining was performed, and resorption areas were observed and scored under an inverted microscope (× 200). Data are means ± SEM for *n* = 3, and one-way ANOVA was performed to compare the results. ^##^*p* < 0.01 compared to untreated cells; *p < 0.05 compared to M-CSF/RANKL-activated cells; ^@^*p* < 0.05 compared to RvD1-stimulated cells

Next, we studied the effects of RvD1 on resorptive activity of human monocyte-derived osteoclasts. As shown by von Kossa staining, a significantly higher erosion surface is observed when cells were M-CSF/RANKL (R-M) differentiated when compared to control, while RvD1 at 500 nM drastically reduces hydroxyapatite matrix erosion to almost background level (*p* < 0.01, Fig. 3a). RvD1 at 100 and 1000 nM inhibits bone resorption in a similar pattern (data not shown). Interestingly, FRP2 silencing by siRNA abolished the inhibitory action of RvD1 (*p* < 0.05).

Then after, additional experiments were conducted using whole mouse femoral bone cultured during 4 weeks. The addition of 500 nM RvD1 reverses bone resorption induced by the R-M treatments to almost baseline level, as observed by hematoxylin and eosin staining (Fig. 3b, *p* < 0.05). This observation is sustained by a reduction in marrow cavity area and an increase in bone surface. Collectively, these data clearly demonstrate the proresolutive and antiresorptive potential of RvD1 on osteoclast differentiation in vitro and in ex vivo.

### RvD1 improves RA clinical endpoints in arthritic mice

**Fig. 4:**
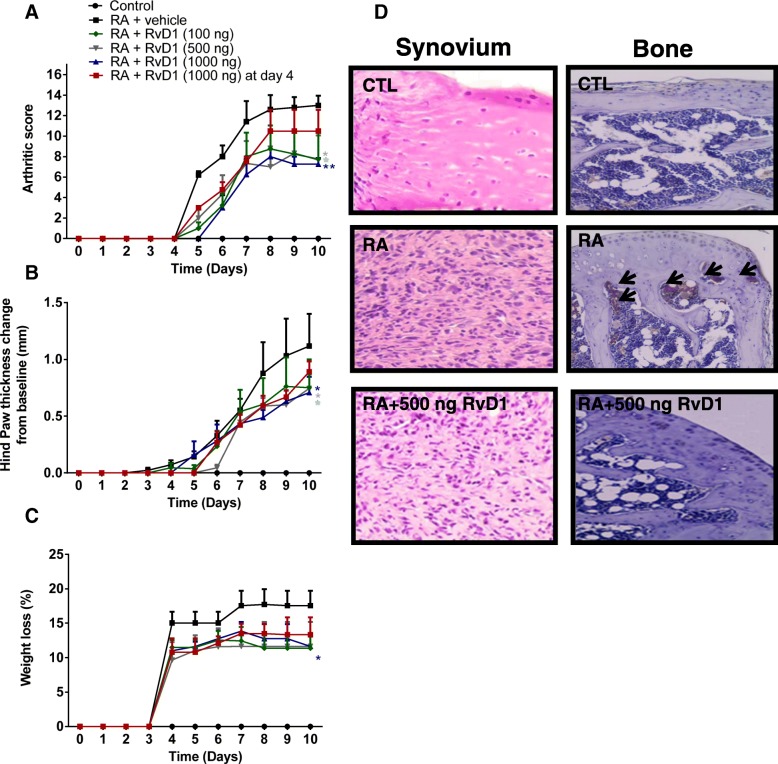
RvD1 treatment improves RA clinical endpoints as well as synovium and bone changes in arthritic mice. Arthritis was induced by i.p. injection of arthritogenic cocktail of five monoclonal anti-type II collagen monoclonal antibodies (mAb) on day 1, followed by i.p. injection of 50 μg LPS on day 4, as described in the “Materials and methods” section. Over the course of the experiment, and for each of the experimental groups (non-immunized control mice, CAIA mice immunized with type II collagen antibody and treated with vehicle, and mice immunized with type II collagen and treated with RvD1 (100, 500, 1000 ng/day) at day 1 or with 1000 ng/ml at day 4, clinical score was attributed by two investigators blinded to groups on a scale of 0–4 for each paw, for a maximum of 16 for all four paws, as described in the “Materials and methods” section (**a**). Hind paw thickness was measured using a caliper and expressed as thickness change from baseline (**b**). Weight evolution was assessed and expressed as percentage of weight loss from baseline (**c**). Data are means ± SEM for *n* = 6, and one-way ANOVA was performed to compare the results. *p < 0.05 and **p < 0.01 compared to RA group. Cell proliferation in synovial membrane and TRAP staining in bone were performed as described in the “Materials and methods” section (**d**)

As expected, CAIA mice develop arthritis on day 4 and exhibit maximum inflammation between days 7 and 10 which was reflected by an increased arthritic score and swelling of paws as well as concomitant weight loss (Fig. 4a–c). All RvD1 treatment groups exhibit a reduced arthritic score when compared to CAIA group (*p* < 0.05, Fig. 4a). Moreover, RvD1 mice had a lesser hind paw thickness over the disease period and reach a maximum effect at the end of the experiment (30% compared to CAIA group, *p* < 0.05, Fig. 4b). Furthermore, CAIA mice exhibit a trend toward weight loss over the disease period as compared to normal mice, with significant (up to 16%) weight loss at the end of the experiment. RvD1 treatment at 500 ng protected the mice from weight loss and reaches a maximum effect at day 10 (30% less than CAIA mice, *p* < 0.05, Fig. 4c). However, no significant differences were found in the measured parameters when 1000 ng RvD1 were given at day 4. Altogether, these findings underscore the clinical therapeutic potential of RvD1 for arthritis management. They indicate that RvD1 treatment clearly alleviates RA main clinical endpoints, suggesting that RvD1 effectively delays RA onset, progression, and severity in an arthritic mouse model.

### RVD1 attenuates synovial proliferation and bone resorption in arthritic mice

This part of the experiment was designed to verify the ability of RvD1 to attenuate histological changes in bone and synovial membrane. As shown in Fig. 4d, RvD1 slightly reduced cell proliferation in synovium and strongly inhibited osteoclast recruitment and activation in arthritic mice. Taken together, these data confirm the in vitro findings and indicate the biological efficacy of RvD1 in suppressing bone and joint damage.

### RvD1 prevents cartilage degradation in knee joints of arthritic mice

**Fig. 5:**
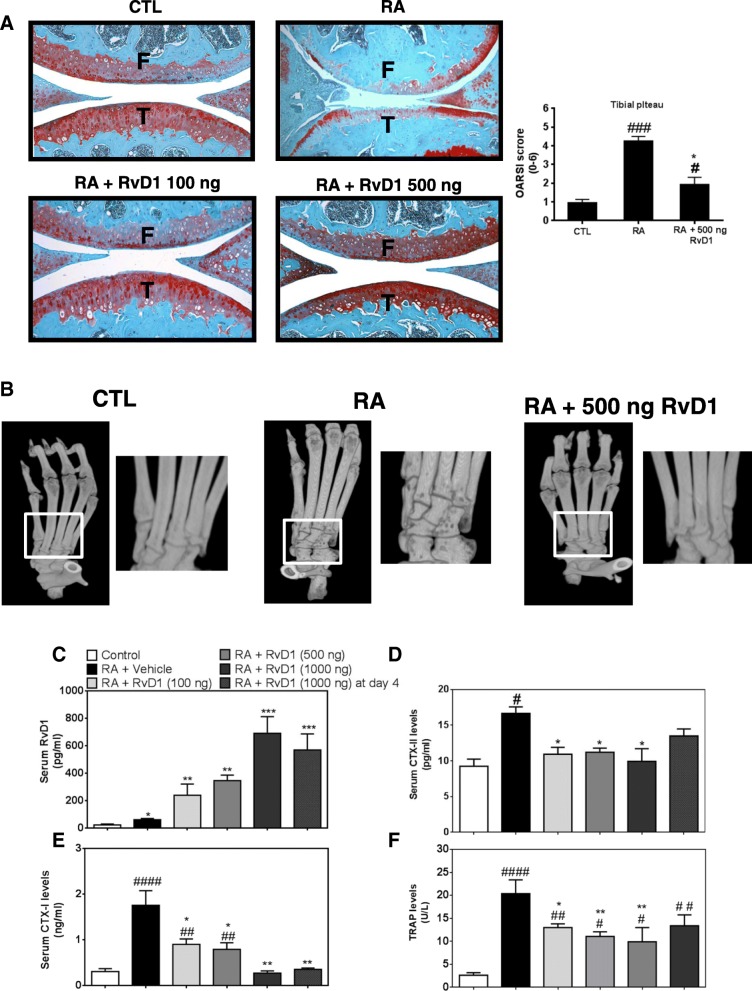
RvD1 daily treatment prevents cartilage and bone alterations in arthritic mouse joints. Arthritis was induced as previously described. After mouse sacrifice, blood was collected. Knees and ankles were removed and then fixed and sectioned as described in the “Materials and methods” section. Knee sections were stained with safranin-O staining and scored (**a**). Ankles were examined with a micro-CT scanner (**b**). Serum RvD1, TRAP, CTX-I, and CTX-II levels were assessed by EIA and ELISA, respectively (**c**–**f**). Data are means ± SEM, and one-way ANOVA was performed to compare the results. ^#^*p* < 0.05, ^##^*p* < 0.01, and ^####^*p* < 0.0001 compared to non-immunized group; *p < 0.05, **p < 0.01, and ***p < 0.001 compared to CAIA group. *n* = 4–5 mice per group. F femur, T tibia

As expected, knee sections from RA mice show signs of histopathologic changes, such as fibrillation with loss of chondrocytes and proteoglycans, as compared to control mice (CTL) (Fig. 5a). In contrast, daily RvD1 treatment (100 and 500 ng) of the arthritic mice significantly reduced cartilage destruction and prevents chondrocytes and proteoglycan loss, when compared to RA, suggesting that RvD1 preserves normal homeostasis of the knee joint. Similar findings were obtained in animals treated with 1000 nM RvD1 given at days 1 and 4 (data not shown). These results are coherent with the arthritic score, and the thickness of the paw that were measured.

### RvD1 inhibits bone destruction in RA mice

We next investigate the ability of RvD1 to prevent bone loss in our RA mice model using μ-CT imaging. μ-CT analyses of the ankles from control, RA mice, and RA mice treated with RvD1 were performed in order to obtain a detailed image of the eroded mineralized bone tissues. As illustrated in Fig. 5b, the intra-peritoneal injection of 500 ng of RvD1 results in a marked reduction of bone erosion across several tarsal bones, as compared to RA mice ankles. These results suggest that RvD1 treatment prevents bone erosion in vivo as our results in vitro.

### RvD1 reduces serum markers of cartilage and bone damage

In order to evaluate by which mechanism RvD1 prevented bone erosion, we investigated the effect of RvD1 administration on the expression of bone destruction markers in RA mouse model. First, our data indicated that RvD1 level increased in serum from RA mice and its administration at different doses enhanced its serum biodisponibility (Fig. 5c). Second, our findings revealed that RvD1 significantly reduced serum cartilage degradation markers, such as CTX-II (Fig. 5d), and serum bone resorption markers, such as CTX-I and TRAP (Fig. 5e, f), as compared to RA mice. Together, these findings suggest the protective effect of RvD1 against cartilage and bone destruction in RA disease and possibly in other bone- and joint-related disorders.

### RvD1 attenuates inflammatory mediators in arthritic mouse serum

**Fig. 6:**
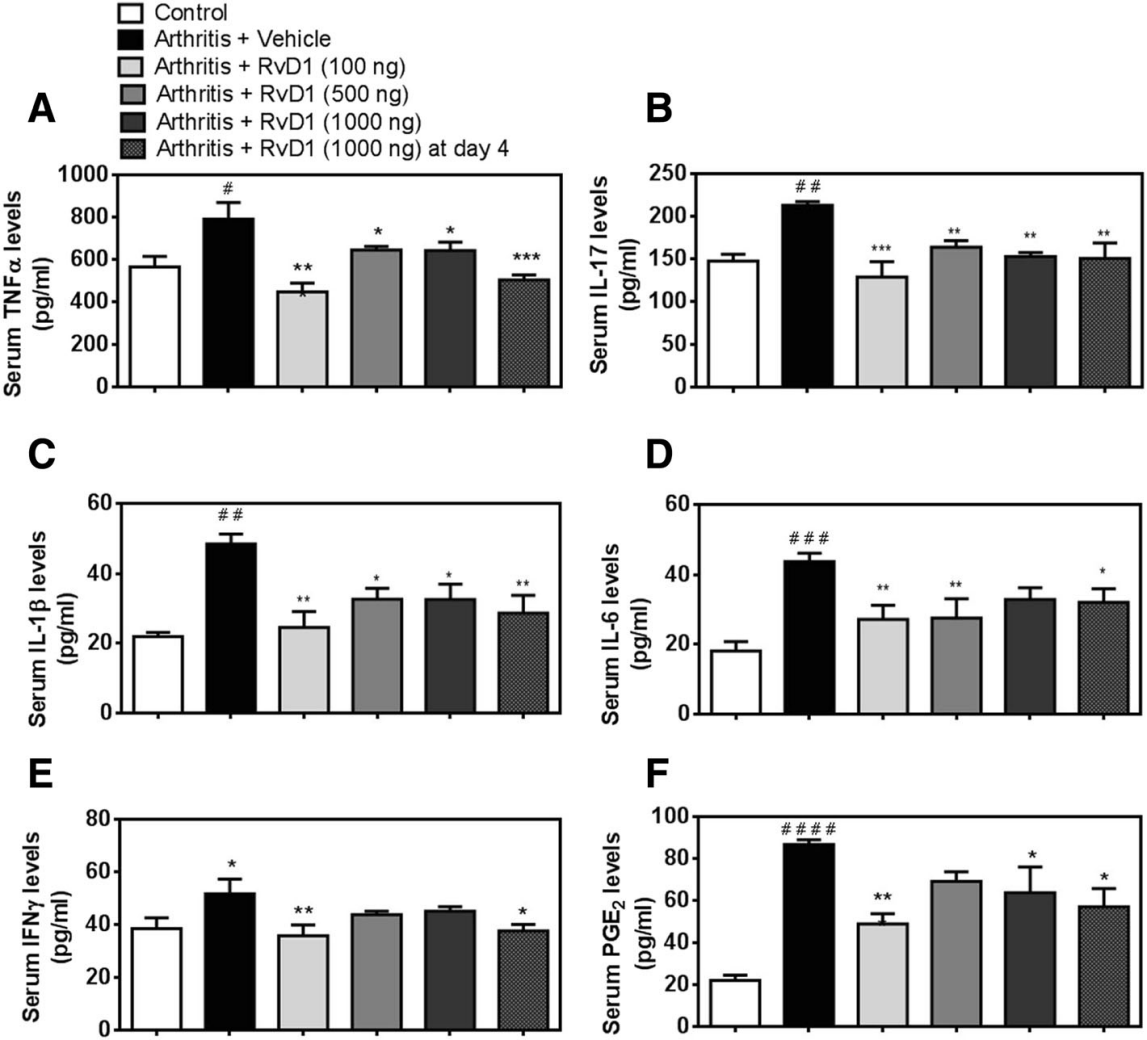
RvD1 daily treatment decreases inflammatory mediators’ release in arthritic mouse serum. Arthritis was induced as previously described in Fig. 4. Blood was collected after mice sacrifice at day 10. Serum levels of inflammatory cytokines (**a**–**e**) as well as PGE_2_ (**f**) were measured by multiplex and EIA assays respectively. Data are means ± SEM. One-way ANOVA was performed to compare the results. *p < 0.05, **p < 0.01, and ***p < 0.001 compared to CAIA group; ^#^*p* < 0.05, ^##^*p* < 0.01, ^###^*p* < 0.001, and ^####^*p* < 0.0001 compared to non-immunized group. *n* = 4–5 mice per group

This part of our study was designed to investigate the anti-inflammatory effect of RvD1 in the CAIA mouse model. As shown in Fig. 6, RvD1 significantly downregulates the production of the most important inflammatory mediators playing crucial roles in RA. In fact, RvD1 given at 100 ng at days 1 and 4 significantly inhibits serum levels of inflammatory cytokines such as TNF-α (Fig. 6a, *p* < 0.01), IL-17 (Fig. 6b, *p* < 0.001), IL-1β (Fig. 6c, *p* < 0.01), IL-6 (Fig. 6d, *p* < 0.01), IFN-γ (Fig. 6e, *p* < 0.01), and PGE_2_ (Fig. 6f, *p* < 0.01) by nearly 50% compared to CAIA group. Altogether, these findings confirm our expectations that RvD1 exerts an anti-inflammatory action in arthritic mice.

## Discussion

As a chronic autoimmune inflammatory condition, RA has been extensively studied. The well-defined and consistent impairment in all RA patients is the severe joint dysfunction, associated with synovial inflammation, cartilage degradation, and osteoporosis [32]. At present, even if some treatments have improved RA therapy, they are still erratic and can cause notable side effects to the patient [33]. Finding a novel therapy could potentially enhance patient quality of life.

In this perspective, we studied an alternative approach for RA therapy using RvD1. RvD1 is an important factor in the resolution phase of inflammation, with well-known pro-resolving and anti-inflammatory properties. We have demonstrated that RvD1 is higher in inflammatory conditions [24], probably in an attempt to reduce the inflammation as an endogenous mechanism. Herein, we describe promising results about the contribution of RvD1 in improving RA symptoms and support the notion that RvD1 prevents arthritic joint disorders, caused by excessive cartilage degradation and bone resorption.

We initially studied the effects of RvD1 on macrophages and osteoclasts, which are key players in promoting and expanding inflammation in RA joint tissues. Macrophages play a pivotal role in promoting and maintaining RA pathogenesis. In concert with chondrocytes and fibroblast-like synoviocytes, they drive cartilage catabolism through the release of catabolic and inflammatory factors. Here, we demonstrated in vitro that RvD1 significantly inhibited osteoclast differentiation and activation as well as the release of pro-inflammatory mediators. In contrast, IL-10, an anti-inflammatory cytokine, was upregulated. Besides, the osteoclast inhibition by RvD1 treatment was associated with TRAP and cathepsin K inhibition. Cathepsin K actions are crucial in normal skeletal physiology [6] and have been implicated in bone and cartilage pathological degradation [34]. In fact, overexpressing cathepsin K in mouse has led to synovitis, as well as cartilage and bone destruction, similar to the manifestation of RA [35]. Otherwise, it has been established that RA serum and synovial fluid present high levels of IL-1β, IL-6, IL-17, and TNF-α, as well as cathepsin K, which triggers osteoclast differentiation and activation in the early phases of the disease [36]. Consistent with our findings, results have indicated that RvD1 reduces osteoclastogenesis through inhibition of the dendritic cell–specific transmembrane protein expression which is essential for cell fusion during osteoclastogenesis [37]. Likewise, DHA showed significant inhibitory effects on osteoclastogenesis, which was reversed by LOX inhibition, suggesting that DHA properties are essentially mediated by RvD1 [37, 38]. Similarly, Boeyens et al. indicated that RANKL-induced and DHA-treated RAW264.7 cells showed suppressed expression of cathepsin K and TRAP [39]. Moreover, Gu et al. showed that RvD1, in addition to other resolution mediators, is a strong inhibitor of pro-inflammatory cytokines produced by monocytes in response to stimulation with LPS [40], while Hsiao et al. suggested that RvD1 increased IL-10 levels in smoke-exposed mouse lungs [41].

In the present study, we also put special emphasis on the potential of RvD1 to prevent bone erosion. Our results demonstrated that RvD1 strongly inhibited hydroxyapatite matrix degradation induced by RANKL + M-CSF using human primary monocytes. Indeed, many authors have reported that TRAP and cathepsin K-positive osteoclasts are largely found in areas of pannus invasion into the bone in RA [5, 42, 43]. Bone damage arises from complex interactions involving osteoclast maturation, mainly mediated via RANKL, M-CSF, and TNF-α pathways [44]. It has been established that RvD1 and its aspirin-triggered epimer switch gene regulation profiles from M1-type pro-inflammatory macrophages into M2-type pro-resolution macrophages involved in homeostasis and bone repair [45, 46]. Hence, our findings contribute to the data dealing about the potential of RvD1 in bone protection.

Furthermore, in order to harness and clarify our findings, we focused on analyzing the involvement of FPR2/ALX receptors in RvD1 effects. Our results demonstrated that FPR2/ALX silencing inhibited RvD1 effects and led to significant osteoclast differentiation as well as important hydroxyapatite matrix erosion, suggesting that RvD1 exerts its actions in part by interacting with FPR2/ALX receptors. These findings are consistent with the literature data indicating that RvD1 actions are mediated by two GPCRs, FPR2/ALX and GPR32 [47]. In fact, Krishnamoorthy et al. have shown that in self-limited peritonitis, RvD1 treatment reduced neutrophil infiltration in human FPR2/ALX-overexpressing transgenic mice, while this effect was not observed in FPR2/ALX knockout mice [48].

In an attempt to refine the in vivo effect of RvD1, another set of experiments was conducted using CAIA mouse model. Our findings showed that daily administration of RvD1 provides its serum bioavailability that is consistent and predictable at every dose. As expected, the arthritic group displayed significant swelling and redness of paws as assessed by macroscopic clinical evaluation and also showed high levels of inflammatory mediators in mouse serum, whereas daily treatment with RvD1 reduced paw swelling and redness and limited mouse weight loss. More importantly, RvD1 strongly reduces TNF-α, IL-17, IL-6, IL-1β, IFN-γ, and PGE_2_, in mouse serum. TNF-α, IL-17, and IL-6 have been shown to be closely correlated with RA activity [36], and IL-1β was involved in cartilage degradation [44].

In accordance with these effects, RvD1 reduced synovial proliferation, osteoclast recruitment, and cartilage destruction in RA mice as depicted by the histological examination. Interestingly, the protective effect of RvD1 in the cartilage and bone was confirmed by the decrease of serum levels of their respective biomarkers, namely CTX-II and CTX-I. Additional findings were obtained on bone resorption, as indicated by μ-CT analysis. Our findings support previous data on cartilage and bone metabolism. In chondrocytes, we demonstrated that RvD1 protects cartilage from degradation and preserves its homeostasis by inhibiting IL-1β-induced matrix metalloproteinase-13 production in human chondrocytes [24].

Additionally, a significant amount of evidence revealed the presence of resolvins, in addition to FPR2/ALX in RA synovial tissues [10, 49, 50]. The interest for RvD1, in general, seems not only due to its potential to neutralize inflammatory and catabolic tissue insults, but also because of increasing data showing that it has the ability to repair injured tissues and most importantly to promote their regeneration. In the context of RA, Norling et al. [10] provided promising finding on RvD1 and its aspirin-triggered epimer. They identified its remarkable efficiency to relieve arthritis symptoms in the K/BxN mouse model. Furthermore, they identified the potential of RvD1 for chondroprotection since it promotes direct cartilage anabolic responses in vitro. Their findings are highly valuable and contribute to the advancement of knowledge on RvD1 and most importantly on how it could be beneficial for RA therapy. The ultimate objective being to build as complete knowledge as possible on a very promising therapy for inflammatory osteoarticular diseases, our study aimed to expand the knowledge by addressing other facets of RvD1 in other important hallmarks of RA. We showed that RvD1 preserves joint structure and prevents bone destruction and erosion by inhibiting osteoclast recruitment.

## Conclusion

In conclusion, the present study demonstrates that RA is indeed positively affected by RvD1 and contributes to other studies clarifying the implication of RvD1 in improving signs and symptoms of RA. This lends support to the notion that RvD1 acts as a potent resolution mediator to control arthritis inflammatory manifestations and may hold promise for use as a potent agent in RA therapy.

## Abbreviations

CTX-I: C-terminal telopeptide type II collagen
CTX-II: C-terminal telopeptide type II collagen
IFN-γ: Interferom-gamma
IL: Interleukin
LDH: Lactate dehydrogenase
LPS: Lipopolysaccharide
M-CSF: Macrophage colony-stimulating factor
NF-κB: Nuclear factor-kappa B
PGE_2_: Prostaglandin E2
RA: Rheumatoid arthritis
RANKL: Receptor activator of NF-κB ligand
RvD1: Resolvin D1
TNF-α: Tumor necrosis factor-alpha
TRAP: Tartrate-resistant acid phosphatase

## References

[1] Scott DL, Wolfe F, Huizinga TW (2010). Rheumatoid arthritis. Lancet.

[2] Fattahi MJ, Mirshafiey A (2012). Prostaglandins and rheumatoid arthritis. Arthritis..

[3] Teng Y, Yin Z, Li J, Li K, Li X, Zhang Y (2017). Adenovirus-mediated delivery of Sema3A alleviates rheumatoid arthritis in a serum-transfer induced mouse model. Oncotarget..

[4] Kiadaliri AA, Felson DT, Neogi T, Englund M (2017). Brief report: rheumatoid arthritis as the underlying cause of death in thirty-one countries, 1987–2011: trend analysis of World Health Organization mortality database. Arthritis Rheumatol..

[5] Dharmapatni AA, Cantley MD, Marino V, Perilli E, Crotti TN, Smith MD, Haynes DR (2015). The X-linked inhibitor of apoptosis protein inhibitor embelin suppresses inflammation and bone erosion in collagen antibody induced arthritis mice. Mediat Inflamm.

[6] Sun P, Liu Y, Deng X, Yu C, Dai N, Yuan X (2013). An inhibitor of cathepsin K, icariin suppresses cartilage and bone degradation in mice of collagen-induced arthritis. Phytomedicine..

[7] Liu Y, Lv J, Yang B, Liu F, Tian Z, Cai Y (2015). Lycium barbarum polysaccharide attenuates type II collagen-induced arthritis in mice. Int J Biol Macromol.

[8] Smolen JS, Landewé R, Breedveld FC, Buch M, Burmester G, Dougados M (2014). EULAR recommendations for the management of rheumatoid arthritis with synthetic and biological disease-modifying antirheumatic drugs: 2013 update. Ann Rheum Dis.

[9] Feldmann M, Maini RN (2015). Perspectives from masters in rheumatology and autoimmunity: can we get closer to a cure for rheumatoid arthritis?. Arthritis Rheumatol..

[10] Norling LV, Headland SE, Dalli J, Arnardottir HH, Haworth O, Jones HR (2016). Pro-resolving and cartilage-protective actions of resolvin D1 in inflammatory arthritis. JCI Insight.

[11] Klarenbeek NB, Güler-Yüksel M, van der Kooij SM, Han KH, Ronday HK, Kerstens PJSM (2011). The impact of four dynamic, goal-steered treatment strategies on the 5-year outcomes of rheumatoid arthritis patients in the BeSt study. Ann Rheum Dis.

[12] Woodworth TG, den Broeder AA (2015). Treating to target in established rheumatoid arthritis: challenges and opportunities in an era of novel targeted therapies and biosimilars. Best Pract Res Clin Rheumatol.

[13] Buckley CD, Gilroy DW, Serhan CN (2014). Proresolving lipid mediators and mechanisms in the resolution of acute inflammation. Immunity..

[14] Serhan CN (2010). Novel lipid mediators and resolution mechanisms in acute inflammation: to resolve or not?. Am J Pathol.

[15] Perretti M, Cooper D, Dalli J, Norling LV (2017). Immune resolution mechanisms in inflammatory arthritis. Nat Rev Rheumatol.

[16] Xu MX, Tan BC, Zhou W, Wei T, Lai WH, Tan JW (2013). Resolvin D1, an endogenous lipid mediator for inactivation of inflammation-related signaling pathways in microglial cells, prevents lipopolysaccharide-induced inflammatory responses. CNS Neurosci Ther.

[17] Corminboeuf O, Leroy X (2014). FPR2/ALXR agonists and the resolution of inflammation. J Med Chem.

[18] Tabas I, Glass CK (2013). Anti-inflammatory therapy in chronic disease: challenges and opportunities. Science..

[19] Luo B, Han F, Xu K, Wang J, Liu Z, Shen Z (2016). Resolvin D1 programs inflammation resolution by increasing TGF-β expression induced by dying cell clearance in experimental autoimmune neuritis. J Neurosci.

[20] Levy BD (2010). Resolvins and protectins: natural pharmacophores for resolution biology. Prostaglandins Leukot Essent Fatty Acids.

[21] Titos E, Rius B, González-Périz A, López-Vicario C, Morán-Salvador E, Martínez-Clemente M (2011). Resolvin D1 and its precursor docosahexaenoic acid promote resolution of adipose tissue inflammation by eliciting macrophage polarization toward an M2-like phenotype. J Immunol.

[22] Xu ZZ, Ji RR (2011). Resolvins are potent analgesics for arthritic pain. Br J Pharmacol.

[23] Oehler B, Mohammadi M, Perpina Viciano C, Hackel D, Hoffmann C, Brack A (2017). Peripheral interaction of resolvin D1 and E1 with opioid receptor antagonists for antinociception in inflammatory pain in rats. Front Mol Neurosci.

[24] Benabdoune H, Rondon EP, Shi Q, Fernandes J, Ranger P, Fahmi H (2016). The role of resolvin D1 in the regulation of inflammatory and catabolic mediators in osteoarthritis. Inflamm Res.

[25] Jovanovic DV, Martel-Pelletier J, Di Battista JA, Mineau F, Jolicoeur FC, Benderdour M (2000). Stimulation of 92-kd gelatinase (matrix metalloproteinase 9) production by interleukin-17 in human monocyte/macrophages: a possible role in rheumatoid arthritis. Arthritis Rheum.

[26] Shi Q, Rondon-Cavanzo EP, Dalla Picola IP, Tiera MJ, Zhang X, Dai K, Benabdoune HA, Benderdour M, Fernandes JC (2018). In vivo therapeutic efficacy of TNFα silencing by folate-PEG-chitosan-DEAE/siRNA nanoparticles in arthritic mice. Int J Nanomedicine.

[27] Vaillancourt F, Silva P, Shi Q, Fahmi H, Fernandes JC, Benderdour M (2011). Elucidation of molecular mechanisms underlying the protective effects of thymoquinone against rheumatoid arthritis. J Cell Biochem.

[28] Lee J, Hong EC, Jeong H, Hwang JW, Kim H, Bae EK (2015). A novel histone deacetylase 6-selective inhibitor suppresses synovial inflammation and joint destruction in a collagen antibody-induced arthritis mouse model. Int J Rheum Dis.

[29] Abusarah J, Benabdoun HA, Shi Q, Lussier B, Martel-Pelletier J, Malo M (2017). Elucidating the role of protandim and 6-gingerol in protection against osteoarthritis. J Cell Biochem.

[30] Pritzker KP, Gay S, Jimenez SA, Ostergaard K, Pelletier JP, Revell PA (2006). Osteoarthritis cartilage histopathology: grading and staging. Osteoarthr Cartil.

[31] Messer JS (2017). The cellular autophagy/apoptosis checkpoint during inflammation. Cell Mol Life Sci.

[32] Hawtree S, Muthana M, Wilson AG (2013). The role of histone deacetylases in rheumatoid arthritis fibroblast-like synoviocytes. Biochem Soc Trans.

[33] Ramiro S, Gaujoux-Viala C, Nam JL, Smolen JS, Buch M, Gossec L (2014). Safety of synthetic and biological DMARDs: a systematic literature review informing the 2013 update of the EULAR recommendations for management of rheumatoid arthritis. Ann Rheum Dis.

[34] Salminen-Mankonen H, Morko J, Vuorio E (2007). Role of cathepsin K in normal joints and in the development of arthritis. Curr Drug Targets.

[35] Morko J, Kiviranta R, Joronen K, Saamanen AM, Vuorio E, Salminen-Mankonen H (2005). Spontaneous development of synovitis and cartilage degeneration in transgenic mice overexpressing cathepsin K. Arthritis Rheum.

[36] Cho YG, Cho ML, Min SY, Kim HY (2007). Type II collagen autoimmunity in a mouse model of human rheumatoid arthritis. Autoimmun Rev.

[37] Yuan J, Akiyama M, Ki N, Sato T, Uematsu H, Morita I (2010). The effects of polyunsaturated fatty acids and their metabolites on osteoclastogenesis in vitro. Prostaglandins Other Lipid Mediat.

[38] Kasonga AE, Deepak V, Kruger MC, Coetzee M (2015). Arachidonic acid and docosahexaenoic acid suppress osteoclast formation and activity in human CD14+ monocytes, in vitro. PLoS One.

[39] Boeyens JC, Deepak V, Chua WH, Kruger MC, Joubert AM, Coetzee M (2014). Effects of ω3-and ω6-polyunsaturated fatty acids on RANKL-induced osteoclast differentiation of RAW264. 7 cells: a comparative in vitro study. Nutrients..

[40] Gu Z, Lamont GJ, Lamont RJ, Uriarte SM, Wang H, Scott DA (2016). Resolvin D1, resolvin D2 and maresin 1 activate the GSK3β anti-inflammatory axis in TLR4-engaged human monocytes. Innate immun.

[41] Hsiao HM, Sapinoro RE, Thatcher TH, Croasdell A, Levy EP, Fulton RA (2013). A novel anti-inflammatory and pro-resolving role for resolvin D1 in acute cigarette smoke-induced lung inflammation. PLoS One.

[42] Schett G (2017). Autoimmunity as a trigger for structural bone damage in rheumatoid arthritis. Mod Rheumatol.

[43] Gravallese EM, Manning C, Tsay A, Naito A, Pan C, Amento E (2000). Synovial tissue in rheumatoid arthritis is a source of osteoclast differentiation factor. Arthritis Rheumatol.

[44] Firestein GS, McInnes IB (2017). Immunopathogenesis of rheumatoid arthritis. Immunity..

[45] Kang JW, Lee SM (2016). Resolvin D1 protects the liver from ischemia/reperfusion injury by enhancing M2 macrophage polarization and efferocytosis. Biochim Biophys Acta.

[46] Schmid M, Gemperle C, Rimann N, Hersberger M (2016). Resolvin D1 polarizes primary human macrophages toward a proresolution phenotype through GPR32. J Immunol.

[47] Krishnamoorthy S, Recchiuti A, Chiang N, Fredman G, Serhan CN (2012). Resolvin D1 receptor stereoselectivity and regulation of inflammation and proresolving microRNAs. Am J Pathol.

[48] Krishnamoorthy S, Recchiuti A, Chiang N, Yacoubian S, Lee CH, Yang R (2010). Resolvin D1 binds human phagocytes with evidence for proresolving receptors. Proc Natl Acad Sci U S A.

[49] Gheorghe KR, Korotkova M, Catrina AI, Backman L, Af Klint E, Claesson HE (2009). Expression of 5-lipoxygenase and 15-lipoxygenase in rheumatoid arthritis synovium and effects of intraarticular glucocorticoids. Arthritis Res Ther.

[50] Hashimoto A, Hayashi I, Murakami Y, Sato Y, Kitasato H, Matsushita R (2007). Antiinflammatory mediator lipoxin A4 and its receptor in synovitis of patients with rheumatoid arthritis. J Rheumatol.

